# Cost-Minimization Analysis for Subcutaneous Daratumumab in the Treatment of Newly Diagnosed Multiple Myeloma in Three Gulf Countries

**DOI:** 10.36469/001c.120288

**Published:** 2024-07-18

**Authors:** Anas Hamad, Shereen Elazzazy, Ruba Y. Taha, Hani Osman, Sana Alblooshi, Islam Elkonaissi, Mustaqeem A. Siddiqui, Khalil Al-Farsi, Mohammed Al Lamki, Sali Emara, Gihan H. Elsisi

**Affiliations:** 1 Hamad Medical Corporation, National Centre for Cancer Care and Research, Doha, Qatar; 2 Tawam Hospital, Abu Dhabi, United Arab Emirates; 3 Sheikh Shakhbout Medical City, Abu Dhabi, United Arab Emirates; 4 Royal Hospital - Ministry of Health, Oman; 5 Sultan Qaboos University Hospital, Oman; 6 Johnson & Johnson Gulf, Dubai, United Arab Emirates; 7 American University in Cairo, Cairo Egypt

**Keywords:** cost-minimization analysis, subcutaneous daratumumab, newly diagnosed multiple myeloma, Gulf countries, Qatar, Oman, United Arab Emirates

## Abstract

**Background:** The second most common hematologic cancer worldwide is multiple myeloma (MM), with incidence and mortality rates that have more than doubled over the past 30 years. The safety and efficacy of daratumumab regimens in the treatment of newly diagnosed MM (NDMM) is demonstrated in clinical trials.

**Objective:** To assess the financial effects of the adoption of subcutaneous daratumumab (dara-SC) rather than intravenous daratumumab (dara-IV) for the treatment of NDMM in three Gulf countries (Qatar, Oman and the United Arab Emirates; UAE), a cost-minimization model was constructed.

**Methods:** We performed static cost minimization analyses from a societal perspective to evaluate the costs and possible reductions in resource utilization associated with a shift from dara-IV infusion to dara-SC injection for NDMM patients over a 5-year time horizon. The model included 2 scenarios: the current scenario in which 100% of patients with NDMM are treated with dara-IV infusion and a future scenario in which dara-SC injection is gradually adopted over the modeled time horizon. The model differentiated precisely between autologous stem cell transplantation (ASCT)–eligible and ASCT-ineligible NDMM patients in terms of their number in each group and the associated therapeutic regimens. One-way sensitivity analyses were also conducted.

**Results:** The model showed that the use of dara-SC in NDMM patients who were eligible or ineligible for ASCT resulted in lower non-drug costs, including premedication drug costs, adverse-effect costs, administration costs, medical staff costs, and indirect costs. The resulting total savings over the 5-year time horizon of the model for Hamad Medical Corporation, Sultan Qaboos University Hospital/Royal Hospital, Sheikh Shakhbout Medical City (SSMC), and Tawam Hospital were QAR −2 522 686, OMR −143 214, AED −30 010 627, and AED −5 003 471, respectively.

**Conclusion:** The introduction of dara-SC as a front-line treatment for NDMM patients in Qatar (Hamad Medical Corporation), Oman (Sultan Qaboos University Hospital, Royal Hospital-MOH), and the UAE (SSMC and Tawam Hospital) can help save resources and minimize constraints on the healthcare system.

## BACKGROUND

The second most common hematologic cancer worldwide is multiple myeloma (MM), with incidence and mortality rates that have more than doubled over the past 30 years.^1,2^ MM treatment strategies focus on the control of active myeloma, the management of complications, and the prevention of disease progression.^3^ Although autologous stem cell transplantation (ASCT), immunomodulatory drugs, proteasome inhibitors (PIs), and combination regimens are available for treating MM patients, MM remains incurable, and patients frequently relapse or become resistant to current therapies.^4,5^ Recently, immunotherapy has changed the paradigm of MM management, as the transmembrane glycoprotein cluster of differentiation 38 (CD38), which is highly expressed in MM cells, is a target for new therapeutic antibodies such as isatuximab and daratumumab.^6^

A retrospective study of 62 MM patients from 2016 to 2018 (40 males, 22 females; median age, 43 years) in a tertiary care institution in the United Arab Emirates (UAE) revealed that 30 of the 62 patients were ASCT-eligible.^7^ In another regional MM burden study that retrieved data from the Global Burden of Disease Study from 1990 to 2019, MM incidence and the number of associated deaths in the UAE and Qatar exhibited the greatest increase over the past 30 years: more than twice that of all other countries.^2^ The age-adjusted incidence rate of MM among Omani males was 1.1, while that among females was 1.5, according to the published 2016 Omani Ministry of Health cancer incidence report.^8^

According to the World Bank, in 2020, current health expenditures as a percentage of gross domestic product in Qatar, the UAE, and Oman were 4.18%, 5.67%, and 5.33%, respectively.^9–12^ Since the early 2000s, the UAE has aspired to establish a program of health system reforms to enhance health services.^13^ Therefore, the UAE is rapidly growing as a healthcare investment destination.^14^ In Qatar, there are high-quality healthcare services, and the current health expenditures per capita value is US $2188.^15,16^ However, there are some challenges concerning the implementation of pharmacovigilance systems, the availability of pharmaceuticals, and the process of medical registration.^17^ In accordance with Al Khalili et al, major healthcare system challenges occurred in Oman due to the COVID-19 pandemic, such as a shortage of experts in risk communication and a scarcity of public health services.^18^

The safety and efficacy of daratumumab regimens in the treatment of ASCT-eligible, newly diagnosed MM (NDMM) patients were demonstrated in 3 clinical trials.^19,20^ In the randomized, open-label, phase 3 CASSIOPEIA trial, 1085 transplant-eligible patients with NDMM who were enrolled at 111 European sites were randomly assigned to receive either 4 pretransplant inductions or 2 posttransplant consolidation cycles of bortezomib, thalidomide, or dexamethasone (VTd) alone or in combination with daratumumab (D-VTd group). The CASSIOPEIA study concluded that the use of the D-VTd regimen before and after ASCT improved the depth of response and progression-free survival with acceptable safety in transplant-eligible NDMM patients.^19^ In the phase II randomized GRIFFIN study, daratumumab in combination with bortezomib, lenalidomide, and dexamethasone (D-VRd) was compared with VRd in 207 transplant-eligible NDMM patients; daratumumab with VRd induction and consolidation improved the depth of response, with no new safety concerns.^21^ In the multicenter, single-arm, phase II MASTER trial, which enrolled 123 NDMM patients with high-risk cytogenetic abnormalities, patients received daratumumab, carfilzomib, lenalidomide, and dexamethasone (D-KRd). The study revealed that D-KRd led to a high rate of negativity for minimal residual disease in NDMM patients.^20^

For transplant-ineligible NDMM patients, the safety and efficacy of daratumumab regimens were also demonstrated in a randomized, open-label, phase III trial that included 733 NDMM ASCT-ineligible patients who were enrolled from March 2015 through January 2017 at 176 sites in 14 countries, where the daratumumab plus lenalidomide and dexamethasone (D-Rd) regimen was tested vs the lenalidomide/dexamethasone regimen. The study concluded that the risk of disease progression or death was significantly lower among those who received DRd than among those who received lenalidomide/dexamethasone alone.^22^

Daratumumab is available in 2 dosage forms: a solution for intravenous (IV) infusion and a solution for subcutaneous (SC) injection.^23,24^ It was reported in a multicenter, open-label, noninferiority, randomized, phase 3 trial that dara-SC was noninferior to dara-IV in terms of efficacy and pharmacokinetics and had an improved safety profile.^25^

### Objective

Increased financial constraints and pressures on healthcare budgets have increased policy makers’ interest in health economic studies and an evidence-based culture around reimbursement for innovative therapies targeted to specific patient populations to achieve the best clinical outcomes in these populations while decreasing total health expenditures.

In this health economic study, we aimed to evaluate the costs and consequences of a shift from intravenous daratumumab (dara-IV) to subcutaneous daratumumab (dara-SC) for NDMM patients from the perspective of representative healthcare systems in Gulf countries, namely, Oman, Qatar, and the UAE.

## METHODS

### Model Structure

Our model was built from the societal perspective in Oman (Royal Hospital-MOH, Sultan Qaboos University Hospital; a tertiary healthcare facility), Qatar (Hamad Medical Corporation; a principal public healthcare provider), and the UAE (Sheikh Shakhbout Medical City (SSMC) and Tawam Hospital; tertiary healthcare facilities in Abu Dhabi). To conduct our health economic study, we performed a systematic literature review to extract all relevant data on resource utilization associated with the treatment of ASCT-eligible and ASCT-ineligible NDMM patients with dara-IV and dara-SC formulations.

Moreover, to fill the gap in knowledge related to local clinical guidelines and practices regarding the treatment of NDMM patients who are eligible for and ineligible for ASCT, we performed interviews with local oncology experts from each institute in the 3 countries mentioned above using a structured questionnaire (**Figure S1**) to validate our model assumptions and inputs. The analysis was reported according to the Consolidated Health Economic Evaluation Reporting Standards (CHEERS) statement.^26^

### Model Design

We performed a static cost minimization analysis (CMA) in Microsoft Excel to evaluate the associated costs and possible reductions in resource utilization associated with shifting NDMM patients from dara-IV infusion to dara-SC injection over a 5-year time horizon. The 2 formulations were proved in clinical trials to be therapeutically equivalent. We chose a static model in our analysis to simplify the calculations and ensure that they can be understood by decision makers. The model included 2 scenarios: the current scenario in which 100% of NDMM patients are treated with dara-IV infusion (represent the current medical practice in all patients in the target population) and a future scenario in which dara-SC injection is gradually adopted over the modeled time horizon.

The model included hypothetical ASCT-eligible and ASCT-ineligible NDMM patients treated at each institute in each country. The ASCT-eligible NDMM patients received either 6 or 8 cycles of daratumumab, 4 of which were given as induction cycles, after which daratumumab was stopped for the administration of high-dose chemotherapy and ASCT. Two or 4 cycles were subsequently administered as consolidation daratumumab cycles. Patients with ASCT-ineligible NDMM received daratumumab until the time of disease progression. The model differentiated precisely between the ASCT-eligible and ASCT-ineligible NDMM patients in terms of the number of patients in each group and the associated therapeutic regimens.

**Figure 1.**
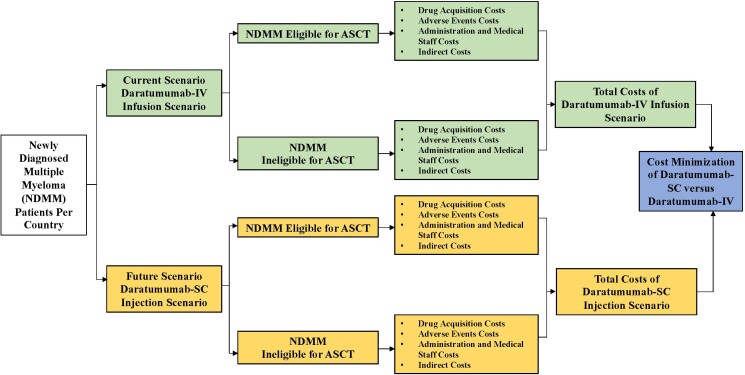
Cost Minimization Model Structure Abbreviations: ASCT, autologous stem cell transplant; IV, intravenous; SC, subcutaneous.

The output of this CMA is the total drug and non-drug costs associated with the treatment of all NDMM patients: ASCT-eligible NDMM patients and ASCT-ineligible NDMM patients receiving dara-IV and dara-SC. The model also showed the possible cost savings between dara-IV and dara-SC. The model structure of our CMA is shown in **Figure 1**.

### Population

The model applied country-specific population characteristics. The model was based on the 2022 population data from the World Bank health plan, with populations of 2 695 122,^27^ 4 576 298,^28^ and 9 441 129^29^ for Qatar, Oman and the UAE, respectively. The numbers of NDMM patients in Qatar (50), Oman (55), and the UAE (60) were obtained from the 2020 World Health Organization Global Cancer Observatory (GLOBOCAN).^30^ For each institute, the percentage of NDMM patients eligible for daratumumab treatment regimens and the proportion of NDMM patients eligible/ineligible for ASCT were validated by a local expert panel from each country. The expert panel in Qatar was composed of 3 experts (1 clinician and 2 health economists) who were affiliated with Hamad Medical Corporation, National Centre for Cancer Care and Research, Doha, Qatar. The expert panel in Oman was composed of 2 experts (1 clinician and 1 health economist) affiliated with the Ministry of Health and Sultan Qaboos University, Oman. The expert panel in the UAE was composed of 4 experts (2 clinicians and 2 health economists) affiliated with Tawam Hospital, Abu Dhabi, UAE and Sheikh Shakhbout Medical City (SSMC), Abu Dhabi, UAE.

These experts were selected from different backgrounds to represent real clinical practices in each country studied. We collected insights from all the experts through 3 rounds of meetings by using the quasi-Delphi panel approach. The experts’ input included insights on the standard practices and treatment courses for these patients within local settings. Furthermore, all assumptions were validated using an expert panel from each country to decrease the uncertainty within the model.

### Treatments

Our model considered different daratumumab treatment regimens for transplant-eligible (2 regimens) and ineligible NDMM patients (3 regimens), pretreatments given prior to dara-IV doses, and treatments for infusion-related reactions (adverse events [AEs]). According to our expert panel, dara-IV infusion is given on average over a 4-hour period, while dara-SC is injected over a 10-minute period.

Pretreatment with dexamethasone 4 mg IV (at Sultan Qaboos University Hospital) or hydrocortisone 100 mg IV (at Hamad Medical Corporation, SSMC, and Tawam Hospital)^31^ was administered to patients 30 minutes prior to dara-IV infusion, and no treatments are given prior to dara-SC injection, according to our expert panel. Grade 3 infusion-related reactions (IRRs) were included in our model based on the literature and validated by our experts, as they occurred more frequently with dara-IV infusions.^25^

For NDMM patients who were transplant-eligible, the first-choice daratumumab regimen for all the institutes was D-VRd. However, the second-choice daratumumab regimen for transplant-eligible NDMM patients in the UAE was D-KRd. For NDMM patients who were transplant-ineligible, the first-choice daratumumab regimen was D-Rd in all 3 countries. In addition, practitioners at Hamad Medical Corporation and Tawam Hospital-administered daratumumab in combination with bortezomib and dexamethasone (D-Vd) as a second-choice regimen. Eventually, Tawam Hospital administered D-VRd as a third-choice regimen for transplant-ineligible NDMM patients. The current and future treatment scenarios for ASCT-eligible and ASCT-ineligible NDMM patients are presented in **Table 1** (sourced from clinical experts). The treatment regimens for NDMM patients who were transplant-eligible or transplant-ineligible are presented in **Table S1**.

**Table 1.** Inputs Applied in the Model

**Parameter**			**Qatar: Hamad Medical Corporation**	**Oman: Sultan Qaboos University Hospital**	**UAE**	
					**SSMC**	**Tawam**
**Model target population**						
Total population 2022 (N)			2 695 122	4 576 298	9 441 129	
NDMM patients (n)			50	55	60	
Eligible for daratumumab (%)			80	100	65	80
NDMM eligible for ASCT (%)			65	60	60	80
NDMM ineligible for ASCT (%)			35	40	40	20
**NDMM eligible for ASCT (% of patients on treatment): Current scenario**						
D-VRd: Daratumumab-IV (1st regimen)			100	100	50	80
D-KRd: Daratumumab-IV (2nd regimen)			0	0	50	20
Total use of regimens			100	100	100	100
**NDMM eligible for ASCT (% of patients on treatment): Future scenario**						
		Year 1	70	40	30	60
		Year 2	60	30	25	50
	Daratumumab-IV	Year 3	50	20	20	40
		Year 4	40	10	15	30
D-VRd		Year 5	30	0	10	20
		Year 1	30	60	20	20
		Year 2	40	70	25	30
	Daratumumab-SC	Year 3	50	80	60	40
		Year 4	60	90	35	50
		Year 5	70	100	40	60
		Year 1			30	16
		Year 2			25	14
	Daratumumab-IV	Year 3			20	10
		Year 4			15	8
D-KRd		Year 5			10	6
		Year 1			20	4
		Year 2			25	6
	Daratumumab-SC	Year 3			60	10
		Year 4			35	12
		Year 5			40	14
**NDMM ineligible for ASCT (% of patients on treatment): Current scenario**						
D-Rd: Daratumumab-IV (1st regimen)	80	100	100	80		
D-Vd: Daratumumab-IV (2nd regimen)	20			10		
D-VRd: Daratumumab-IV (3rd regimen)				10		
Total	100	100	100	100		
**NDMM ineligible for ASCT (% of patients on treatment): Future scenario**						
		Year 1	56	40	60	60
		Year 2	48	30	50	50
	Daratumumab-IV	Year 3	40	20	40	40
D-Rd		Year 4	32	10	30	30
		Year 5	24	0	20	20
		Year 1	24	60	40	20
	Daratumumab-SC	Year 2	32	70	50	30
		Year 3	40	80	60	40
**NDMM ineligible for ASCT (% of patients on treatment): Future scenario**						
D-Rd	Daratumumab-SC	Year 4	48	90	70	50
		Year 5	56	100	80	60
		Year 1	14			8
		Year 2	12			7
	Daratumumab-IV	Year 3	10			6
		Year 4	8			5
D-Vd		Year 5	6			4
		Year 1	6			2
		Year 2	8			3
	Daratumumab-SC	Year 3	10			4
		Year 4	12			5
		Year 5	14			6
		Year 1				8
		Year 2				7
	Daratumumab-IV	Year 3				6
		Year 4				5
D-VRd		Year 5				4
		Year 1				2
		Year 2				3
	Daratumumab-SC	Year 3				4
		Year 4				5
		Year 5				6

Abbreviations: ASCT, autologous stem cell transplantation; D-KRd, daratumumab, carfilzomib, lenalidomide, and examethasone; D-Rd, daratumumab plus lenalidomide and dexamethasone; D-Vd, daratumumab in combination with bortezomib and dexamethasone; D-VRd, daratumumab in combination with bortezomib, lenalidomide, and dexamethasone; IV, intravenous; NDMM, newly diagnosed multiple myeloma; SC, subcutaneous; SSMC, Sheikh Shakhbout Medical City.

### Staff Working Time per Dose

According to our expert panel, the oncology pharmacist is responsible for the preparation of the daratumumab dose. The pharmacist needs 90 to 120 minutes on average to prepare the dara-IV infusion, while preparing the dara-SC injection requires only 2 minutes.

The nurse is responsible for preparing the IV sets for the IV infusion of daratumumab to patients; this process takes approximately 30 minutes, while no time is needed for the nurse to prepare the dara-SC. In addition, the nurse is responsible for checking patients during the IV infusion of daratumumab.

It was assumed that the number of working days per year is 264 days (5 days/week) and that the number of working hours per day is 8 hours.

### Costs and Healthcare Resource Use

Our health economic model included both direct and indirect costs associated with dara-IV infusion and dara-SC injection. All direct medical costs were presented in country-specific currency (Qatar, Qatari Riyal [QAR]; Oman, Omani Riyal [OM]; UAE, Arab Emirates Dirham [AED]). The unit costs were obtained from the 2023 price lists of Hamad Medical Corporation, Sultan Qaboos University Hospital, SSMC Hospital, and Tawam Hospital (**Table 2**).

**Table 2.** Costs Measured in the Model

**Cost Parameters**		**Qatar (QAR): Hamad Medical Corporation**	**Oman (OMR): Sultan Qaboos University Hospital**	**UAE (AED)**	
				**SSMC**	**Tawam Hospital**
**Drug Costs**	**Dose**		**Base Case (Low, High Value)**		
Daratumumab (price/ vial)	IV 400 mg	6572 (5258, 7887)	722 (578, 866)	6305 (5044, 7566)	6500 (5200, 7800)
	SC 1800 mg	19 699 (15 759, 23 639)	1976 (1581, 2371)	22 165 (17 732, 26 598)	22 500 (18 000, 27 000)
Prednisone (price/tablet)	20 mg	0.2 (0.16, 0.25)	0.4 (0.3, 0.5)	0.4 (0.3, 0.5)	0.5 (0.4, 0.6)
	25 mg	991 (793, 1189)	16 (13, 20)	827 (662, 992)	900 (720, 1080)
Lenalidomide (unit price)	15 mg	1100 (880, 1320)			800 (640, 960)
	10 mg	1099 (879, 1319)		688 (551, 826)	700 (560, 840)
	5 mg	1056 (844, 1267)	16 (13, 20)	658 (526, 790)	600 (480, 720)
Dexamethasone (price/ injection)	4 mg		0.4 (0.3, 0.5)	3 (2,4)	2 (1.6, 2.4)
	8 mg	1 (0.8, 1.2)			
Bortezomib (price/ injection)	3.5 mg	3252 (2602, 3903)	57 (46, 68)	783 (627, 940)	675 (540, 810)
Thalidomide (unit price)	50 mg				50 (40, 60)
Carfilzomib (price/ injection)	60 mg			4796 (3837, 5755)	5500 (4400, 6600)
	10 mg		0.4 (0.3, 0.5)	1 (0.7,1.1)	
Chlorphenamine (price/ injection)	2 mg	2 (1.36, 2.04)			
	50 mg				5 (4, 6)
Montelukast (price/ tablet)	10 mg	3 (2,4)	0.4 (0.3, 0.5)	1 (0.7, 1.01)	1 (0.8, 1.2)
Paracetamol (price/ injection)	100 mg		0.4 (0.3, 0.5)		5 (4, 6)
	10 mg/1 ml (100 ml)	3 (2.6, 3.9)		4 (2.9, 4.3)	
	100 mg (price/ injection)	3 (2.1, 3.1)			10 (8, 12)
Hydrocortisone					
	10 mg (price/ tablet)			0.4 (0.3,0.42)	
**Unit Non-drug Costs**		**Base Case (Low, High Value)**			
Syringe	3 (2.1, 3.2)	0.4 (0.3, 0.5)	0.3 (0.2, 0.4)	0.6 (0.5, 0.7)	
One pair of gloves	3 (2.6, 3.9)	0.4 (0.3, 0.5)	0.1 (0.09, 0.13)	0.6 (0.5, 0.7)	
Normal saline IV	4 (2.8, 4.2)	0.4 (0.3, 0.5)	3 (2.7, 4)	4 (3, 5)	
IV set: Line insertion	22 (18, 26)	1 (0.9, 1.4)	8 (6, 9)	950 (760, 1140)	
Alcohol swab	0.02 (0.017, 0.03)	0.4 (0.3, 0.5)	0.017 (0.014, 0.02)	1 (0.8, 1.2)	
Cannula	20 (16, 24)	0.4 (0.3, 0.5)	20 (16, 24)	11 (8.8, 13.2)	
Torniques-elastic	0.18 (0.14, 0.22)	0.4 (0.3, 0.5)	10 (8, 12)	5 (4, 6)	
Tegaderm dressing	2 (1.8, 2.6)	1.1 (0.9, 1.4)	2 (1.9, 2.8)	1 (0.8, 1.2)	
Pharmacist average monthly salary	20 000 (16 000, 24 000)	1140 (912, 1368)	25 000 (20 000, 30 000)	19 600 (15 680, 23 520)	
Nurse average monthly salary	15 000 (12 000, 18 000)	950 (760, 1140)	15 000 (12 000, 18 000)	18 500 (14 800, 22 200)	
Hospitalization day	7500 (6000, 9000)	30 (24, 36)	30 939 (24 751, 37 126)	10 000 (8000, 12 000)	
Day care	5400 (4320, 6480)				
Hospital meal		2 (1.5, 2.3)			
**Indirect Cost**		**Base Case (Low, High Value)**			
NDMM, ASCT- ineligible: Working (%)	20% (16%, 24%)	40% (32%, 48%)	25% (20%, 30%)	20% (16%, 24%)	
NDMM: ASCT- eligible: Working (%)	60% (48%, 72%)	100% (80%, 120%)	80% (64%, 96%)	90% (72%, 108%)	
GDP per capita	320 489 (256 391, 384 587)	9634 (7707, 11 561)	197 243 (157 794, 236 692)		

Abbreviations: ASCT, autologous stem cell transplantation; IV, intravenous; NDMM, newly diagnosed multiple myeloma; SC, subcutaneous; SSMC, Sheikh Shakhbout Medical City.

The direct medical costs included in our model were drug acquisition costs, dara-IV infusion preparation costs, dara-SC injection preparation costs, AE treatment costs, hospitalization and hospital daily care costs, and medical staff salaries (**Table 2**).

The resources used for the preparation and administration of dara-IV included syringes, gloves, normal saline, IV sets, alcohol swabs, cannulas, tourniquets, normal saline flush, Tegaderm dressing, and a meal offered to patients on dara-IV infusion, while those for dara-SC included syringes, gloves and alcohol swabs only.

The indirect costs (loss of productivity) included in our model were assumed to be 1 working day for dara-IV patients due to the time spent by the patients waiting for IV-set preparation, the infusion time and the post-IV infusion administration observation time needed. The percentages of ASCT-eligible and ASCT-ineligible NDMM patients who were working in each country were obtained from our expert panel. The average wage/day in our model was calculated based on the published GDP per capita for each country in the 2022 figures from the World Bank.^32,33^

### Sensitivity Analyses

Deterministic sensitivity analysis (DSA) was conducted to test the model uncertainties and assumptions connected to the study inputs, to assure the robustness of the cost minimization model and to evaluate the model inputs that have a significant impact on the results. We varied the inputs between lower and upper values of ±20%, and we presented the DSA in tornado diagrams, which illustrated the most sensitive input values affecting the model results.

## RESULTS

### Base Case

The base case results of our model are presented in **Table 3** (detailed full results are presented in **Table S2**). The model showed that the use of dara-SC in NDMM patients who were both eligible for and ineligible for ASCT resulted in lower non-drug costs, including premedication drug costs, AE costs, administration costs, medical staff costs, and indirect costs.

**Table 3.** Annual Cost Results in Current and Future Model Scenarios

**Cost**			**Qatar (QAR): Hamad Medical Corporation**	**Oman (OMR): Sultan Qaboos University Hospital**	**UAE (AED)**	
					**SSMC**	**Tawam Hospital**
Year 1	Drug costs	Current scenario	24 044 938	2 416 962	28 738 746	31 126 646
		Future scenario	24 993 859	2 434 518	29 490 067	32 173 507
		Savings	948 922	17 556	751 322	1 046 861
	Non-drug medical costs	Current scenario	6 118 572	122 692	32 885 306	13 493 604
		Future scenario	4 347 299	52 087	20 117 310	10 403 571
		Savings	-1 771 272	-70 606	-12 767 996	-3 090 033
	Total costs	Current scenario	30 163 509	2 539 654	61 624 052	44 620 250
		Future scenario	29 341 159	2 486 605	49 607 377	42 577 078
		Savings	-822 350	-53 050	-12 016 675	-2 043 172
Year 2	Drug costs	Current scenario	16 239 941	1 227 269	11 370 399	15 584 525
		Future scenario	16 786 588	1 236 588	11 647 876	16 099 699
		Savings	546 646	9 319	277 477	515 174
	Non-drug medical costs	Current scenario	2 737 776	55 797	9 716 101	4 310 351
		Future scenario	1 681 029	18 337	5 000 654	2 765 507
		Savings	-1 056 747	-37 460	-4 715 447	-1 544 844
	Total costs	Current scenario	18 977 717	1 283 065	21 086 500	19 894 876
		Future scenario	18 467 616	1 254 925	16 648 530	18 865 207
		Savings	-510 101	-28 141	-4 437 970	-1 029 669
Year 3	Drug costs	Current scenario	5 858 232	660 774	7 637 826	4 402 445
		Future scenario	6 212 540	666 860	7 879 987	4 660 032
		Savings	354 308	6 086	242 161	257 587
	Non-drug medical costs	Current scenario	1 419 587	31 884	7 066 255	1 657 827
		Future scenario	734 659	7 420	2 950 956	885 406
		Savings	-684 929	-24 464	-4 115 299	-772 422
	Total costs	Current scenario	7 277 819	692 658	14 704 081	6 060 272
		Future scenario	6 947 199	674 281	10 830 943	5 545 438
		Savings	-330 621	-18 378	-3 873 138	-514 835
Year 4	Drug costs	Current scenario	5 858 232	660 774	7 637 826	4 402 445
		Future scenario	6 283 401	667 621	7 920 347	4 724 429
		Savings	425 169	6 847	282 522	321 984
	Non-drug medical costs	Current scenario	1 419 587	31 884	7 066 255	1 657 827
		Future scenario	597 673	4 362	2 265 073	692 300
		Savings	-821 914	-27 522	-4 801 182	-965 527
	Total costs	Current scenario	7 277 819	692 658	14 704 081	6 060 272
		Future scenario	6 881 074	671 983	10 185 420	5 416 729
		Savings	-396 745	-20 675	-4 518 661	-643 543
Year 5	Drug costs	Current scenario	5 858 232	660 774	7 637 826	4 402 445
		Future scenario	6 354 263	668 382	7 960 708	4 788 826
		Savings	496 031	7 608	322 882	386 381
	Non-⁠drug medical costs	Current scenario	1 419 587	31 884	7 066 255	1 657 827
		Future scenario	460 687	1 304	1 579 190	499 195
		Savings	-958 900	-30 580	-5 487 066	-1 158 633
	Total costs	Current scenario	7 277 819	692 658	14 704 081	6 060 272
		Future scenario	6 814 950	669 686	9 539 897	5 288 020
		Savings	-462 869	-22 972	-5 164 184	-772 252

The total drug costs of the current and future scenarios for Hamad Medical Corporation, Sultan Qaboos University Hospital, SSMC and Tawam Hospital were QAR 57 859 575 and QAR 60 630 651; OMR 5 626 554 and OMR 5 673 970; AED 63 022 622 and AED 64 898 985; and AED 59 918 505 and AED 62 446 493, respectively. The nondrug costs were QAR 13 115 110 and QAR 7 821 347; OMR 274 140 and OMR 83 510; AED 63 800 173 and AED 31 913 182; and AED 22 777 437 and AED 15 245 979 for the same hospitals for the current and future scenarios, respectively. The resulting total savings over the 5-year time horizon of the model for Hamad Medical Corporation, Sultan Qaboos University Hospital, SSMC and Tawam Hospital were QAR −2 522 686, OMR −143 214, AED −30 010 627 and AED −5 003 471, respectively.

### Sensitivity Analysis

**Figure 2.**
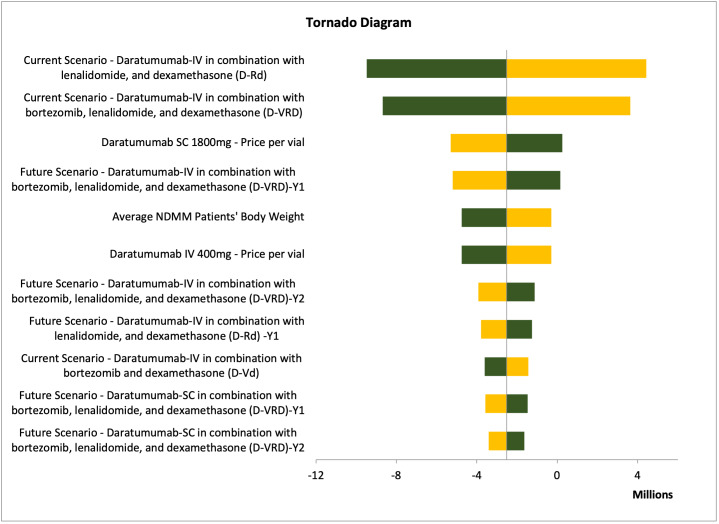
Results of One-way Sensitivity Analysis of Hamad Medical Corporation, Qatar The orange segment indicates the low value of the output, while the green segment indicates the high value of the output. Abbreviations: D-Rd, daratumumab with lenalidomide, and dexamethasone; D-Vd, daratumumab with bortezomib and dexamethasone; D-VRD, daratumumab with bortezomib, lenalidomide, and dexamethasone; IV, intravenous; NDMM, newly diagnosed multiple myeloma; SC, subcutaneous.

**Figure 3.**
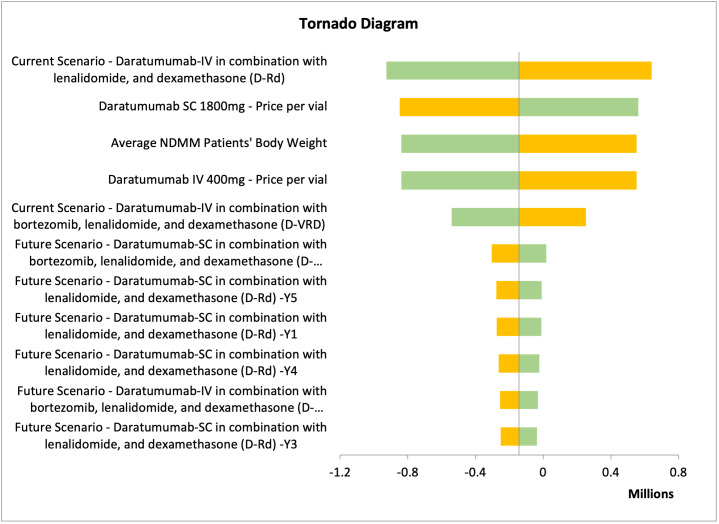
Results of One-way Sensitivity Analysis of Sultan Qaboos University, Oman The orange segment indicates the low value of the output, while the green segment indicates the high value of the output. Abbreviations: D-Rd, daratumumab with lenalidomide, and dexamethasone; D-Vd, daratumumab with bortezomib and dexamethasone; D-VRD, daratumumab with bortezomib, lenalidomide, and dexamethasone; IV, intravenous; NDMM, newly diagnosed multiple myeloma; SC, subcutaneous.

One-way sensitivity analyses were performed to assure the robustness of the results. The different parameters varied by 10% to 20% above or below their base case values. The parameters tested were the clinical parameters, dosage regimens, drug acquisition costs, service costs, and productivity costs for each treatment arm. For Hamad Medical Corporation, Sultan Qaboos University Hospital, and Tawam Hospital, the most impactful parameter affecting the results was the treatment approach for the current scenario: dara-IV in combination with lenalidomide and dexamethasone (**Figures 2 and 3**; **Figure S1**). In contrast, for SSMC, the number of NDMM ineligible patients receiving daratumumab was the most impactful parameter (**Figure S2**).

## DISCUSSION

Patients with MM who receive dara-IV infusion must endure an extended infusion time and may experience infusion-related events; both experiences impair patient quality of life (QoL). Subcutaneous administration of daratumumab is anticipated to be more manageable and to result in fewer AEs than dara-IV after administration.^25^ Based on best clinical practice, the first dose of dara-IV is infused over 7 hours, and subsequent doses are infused over 3 to 4 hours.^23,25^ The prolonged infusion time of dara-IV affects both patient QoL and healthcare system resources.^34^

According to our cost-minimization model, the introduction of dara-SC to the treatment regimens for both ASCT-eligible and ASCT-ineligible NDMM patients at the Hamad Medical Corporation, Sultan Qaboos University Hospital, SSMC, and Tawam Hospital resulted in profound cost savings in terms of premedication drug costs, AE costs, administration costs, medical staff costs, and indirect costs. These results may indicate that shifting NDMM patients to dara-SC formulations can decrease the pressure on hospital staff members.

Some of the implications on the healthcare system and society of dara-SC are both shortening the prolonged IV infusion time (daratumumab injection is given over only 3-5 minutes) and decreasing the risk of IRRs.^24,25^ The decrease in the rate of IRRs with dara-SC was an approximately 3-fold reduction compared with dara-IV infusion.^35^ Thus, dara-SC has decreased strain on healthcare system resources owing to its simplified drug preparation and administration, which also contributed to a decrease in the number of medication/preparation errors.^25,36^

To decrease the strain on healthcare facilities, most facilities are implementing the rapid daratumumab infusion protocol (after the first 2 doses), in which the drug is given IV over a 90-minute period instead of its standard infusion time of 3 to 4 hours.^37,38^ This rapid infusion protocol provides cost savings in United States^37^ but may increase the risk of IRRs.^39^

Dara-SC has been proven safe in real-world clinical practice based on a retrospective study that included data from August 2020 until November 2020 for 58 patients. That study found that dara-SC was extremely well tolerated and could be safely administered without the need for monitoring or rescue medications at home.^35^ These real-world data on the safety and tolerability of dara-SC and the decreased injection time of this formulation are very important during pandemics and in the era of climate change, as many safety measures are oriented at reducing the amount of time spent at infusion centers to decrease the patient’s risk of infection.^35^ Within this context and based on clinical trials that included ASCT-ineligible NDMM patients, dara-SC as a monotherapy or in addition to lenalidomide/dexamethasone was safe and preferred over dara-IV in these patients.^40^

A study that analyzed the clinical administration approach for dara-IV vs dara-SC for 802 MM patients who received treatment at Mayo Clinic infusion centers reported that the median chair time and median clinic time were reduced with dara-SC, the need for post-administration medications was reduced for MM patients receiving dara-SC compared with dara-IV, and reactions related to drug administration were rarer in dara-SC MM patients. That study concluded that the observed reduction in clinic times with the use of dara-SC in MM patients might indicate that this treatment approach could result in time savings and thus free up clinic resources.^41^

In another study that evaluated the benefits of switching from dara-IV to dara-SC from the perspective of healthcare providers in the United Kingdom, the switch from dara-IV to dara-SC was found to be beneficial to patients and healthcare providers, as it simplified treatment, reduced pressure on hospitals, and improved patients’ QoL.^42^

Several health economic studies evaluated the cost and time savings achieved when using dara-SC as a treatment regimen for MM patients. An Italian budget impact study tested the monetary impact of switching Italian MM patients from dara-IV to dara-SC and reported that switching 95% of MM patients to dara-SC resulted in resource savings for all regimens considered and in every cost category within the Italian healthcare system.^43^ Another Italian cost-minimization study testing the effect of shifting MM patients from dara-IV to dara-SC showed that switching MM patients to the SC formulation could save resources in Italian healthcare settings.^44^

In a study evaluating the budgetary impact of replacing dara-IV with dara-SC over a 5-year period from the payer perspective in Sweden, substantial healthcare cost savings occurred over the 5-year model period due to shifting MM patients from dara-IV to dara-SC.^45^

Our study has several strengths. First, it is the first cost-minimization analysis to evaluate the introduction of dara-SC to NDMM treatment regimens in the healthcare system in Gulf countries. Furthermore, to decrease the uncertainty in our model, a carefully designed sensitivity analysis was conducted to guarantee the robustness of the model. In addition, we validated all the inputs and assumptions in our analysis through our expert panel.

This study also has the following limitations. We obtained clinical data on the treatment of NDMM patients and the target patients’ characteristics (average body weight and average body surface area) from our expert panels at Hamad Medical Corporation, Sultan Qaboos University Hospital, SSMC, and Tawam Hospital due to a lack of published data. In addition, we considered all NDMM patients in our model to be progression-free, and we did not introduce any second-line treatments for patients in the model to focus on the costs of daratumumab as a first-line treatment for MM. Furthermore, we did not consider vial waste for dara-IV, although this might have decreased the total drug costs of dara-IV in our analysis. Finally, we did not calculate administration costs, AE costs, or staff working hour costs for other treatments within each daratumumab regimen and instead calculated only the costs associated with daratumumab use.

## CONCLUSION

The introduction of dara-SC as a front-line treatment for NDMM patients in Qatar (Hamad Medical Corporation), Oman (Sultan Qaboos University Hospital, Royal Hospital-MOH), and the UAE (SSMC and Tawam Hospital) can help save resources and minimize constraints on the healthcare system.

## Supplementary Material

Online Supplementary Material
