# Supplementary material for: Cost-Minimization Analysis for Subcutaneous Daratumumab in the Treatment of Newly Diagnosed Multiple Myeloma in Three Gulf Countries
Source: J Health Econ Outcomes Res. 2024 Jul 18;11(2):9–19. doi: 10.36469/001c.120288 (PMC11260124; doi:10.36469/001c.120288)
Supplement: Online Supplementary Material [file jheor_2024_11_2_120288_237185.pdf]

Journal of Health Economics and Outcomes Research

Online Supplementary Material

Cost-Minimization Analysis for Subcutaneous Daratumumab in the Treatment of Newly Diagnosed Multiple Myeloma in 3 Gulf countries. *JHEOR*. 2024;11(2):9-19. doi:10.36469/jheor.2024.120288

Figure S1: Results of One-way Sensitivity Analysis of Tawam, Emirates

Figure S2: Results of One-way Sensitivity Analysis of SSMC, Emirates

This supplementary material has been provided by the authors to give readers additional information about their work.

This is an open-access article distributed under the terms of the Creative Commons Attribution 4.0 International License (CCBY-4.0). View this license's legal deed at http://creativecommons.org/licenses/by/4.0 and legal code at http://creativecommons.org/licenses/by/4.0/legalcode for more information.

Figure S1. Results of One-way Sensitivity Analysis of Tawam, Emirates

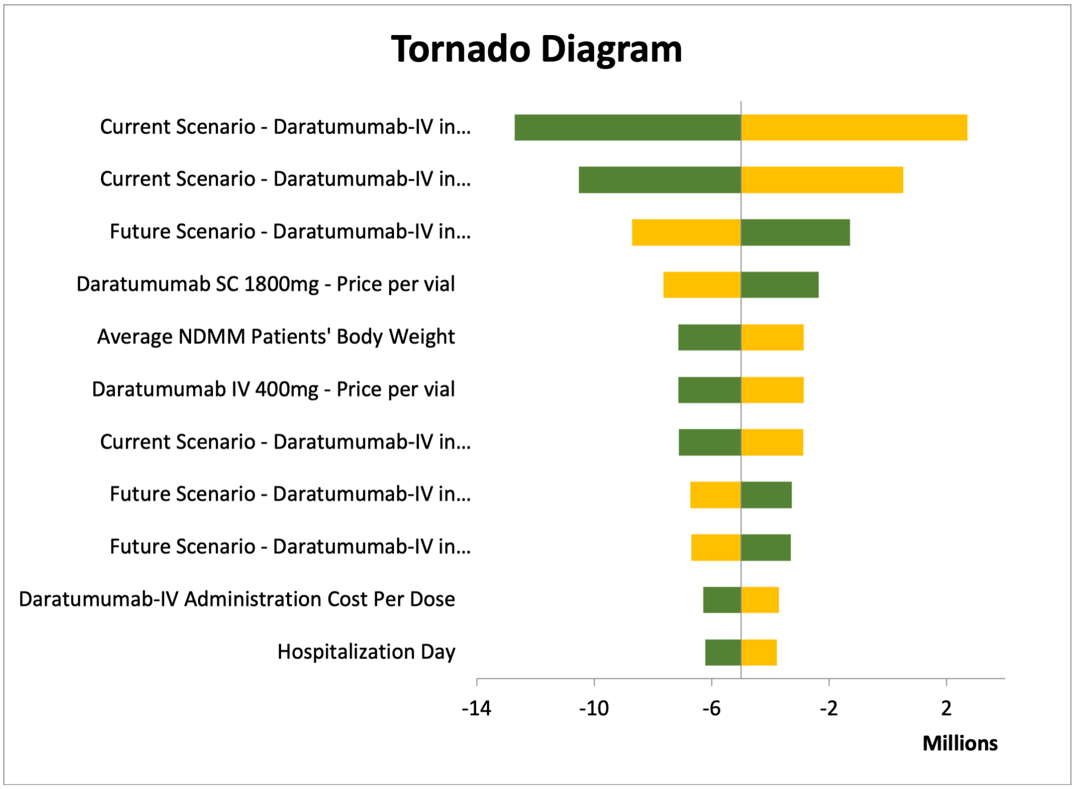

The orange segment indicates the low value of the output, while the green segment indicates the high value of the output.
Abbreviations: IV, intravenous; SC, subcutaneous.

Figure S2. Results of One-way Sensitivity Analysis of SSMC, Emirates

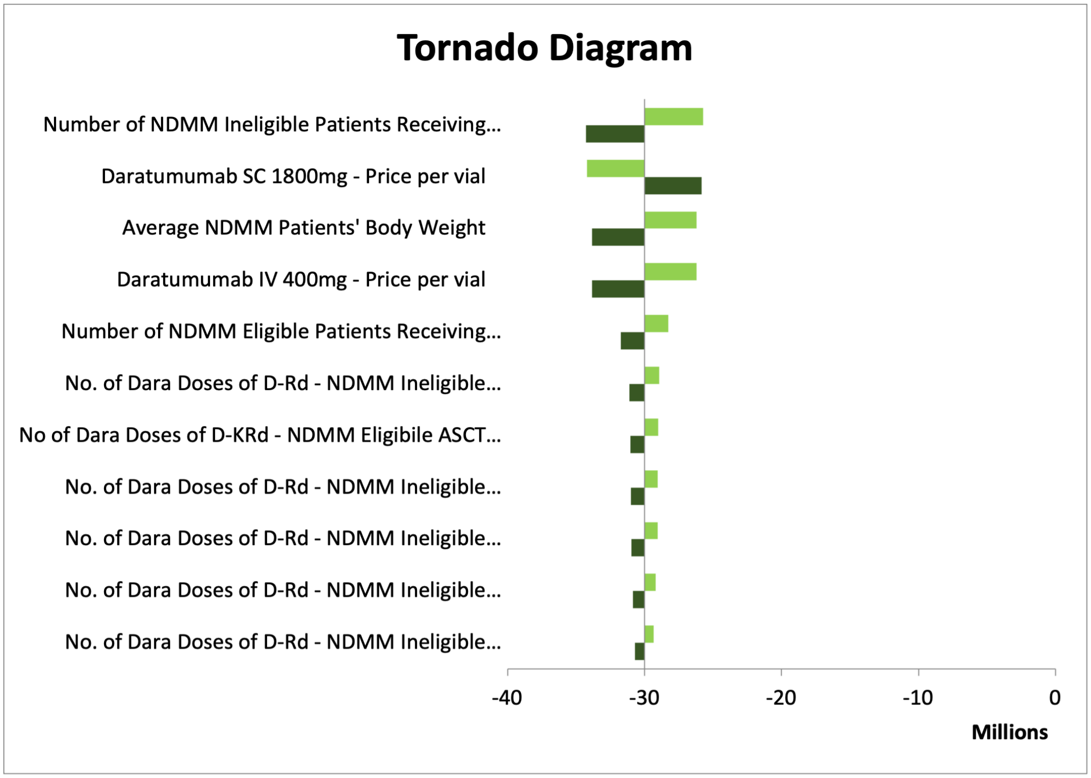

The light green segment indicates the low value of the output, while the dark green segment Abbreviations: D-KRd, daratumumab with carfilzomib, lenalidomide, and dexamethasone; D-Rd, daratumumab with lenalidomide and dexamethasone; D-Vd, daratumumab with bortezomib and dexamethasone; D-VRd, daratumumab with bortezomib, lenalidomide, and dexamethasone; NDMM, newly diagnosed multiple myeloma.
